# Association between behavioral and psychological symptoms and cerebral small vessel disease MRI findings in memory clinic patients

**DOI:** 10.3389/fnagi.2023.1143834

**Published:** 2023-03-24

**Authors:** Masaki Shinohara, Kana Matsuda, Yuichiro Ii, Ken-ichi Tabei, Naoko Nakamura, Yoshinori Hirata, Hidehiro Ishikawa, Hirofumi Matsuyama, Keita Matsuura, Masayuki Maeda, Hidekazu Tomimoto, Akihiro Shindo

**Affiliations:** ^1^Department of Neurology, Mie University Graduate School of Medicine, Tsu, Japan; ^2^Department of Dementia Prevention and Therapeutics, Mie University Graduate School of Medicine, Tsu, Japan; ^3^School of Industrial Technology, Advanced Institute of Industrial Technology, Tokyo Metropolitan Public University Corporation, Tokyo, Japan; ^4^Department of Neuroradiology, Mie University Graduate School of Medicine, Tsu, Japan

**Keywords:** small vessel disease, mild cognitive impairment, dementia, microbleeds, behavioral psychological symptoms of dementia

## Abstract

**Objectives:**

Cerebral small vessel disease (SVD) is commonly observed among elderly individuals with cognitive impairment and has been recognized as a vascular contributor to dementia and behavioral and psychological symptoms (BPS), however, the relationship between BPS and SVD burden remains unclear.

**Methods:**

We prospectively recruited 42 patients with mild cognitive impairment (MCI) or mild dementia from the memory clinic in our hospital, who were assigned to either a clinical dementia rating (CDR) of 0.5 or 1.0, respectively. The presence of BPS was determined through interviews with caregivers. The patients underwent brain MRI and three types of SVD scores, total, cerebral amyloid angiopathy (CAA), and modified CAA, were assigned. Patients were also evaluated through various neuropsychological assessments.

**Results:**

The CDR was significantly higher in patients with BPS (*p* = 0.001). The use of antihypertensive agents was significantly higher in patients without BPS (*p* = 0.038). The time taken to complete trail making test set-A was also significantly longer in patients with BPS (*p* = 0.037). There was no significant difference in total SVD and CAA-SVD score (*p* = 0.745, and 0.096) and the modified CAA-SVD score was significantly higher in patients with BPS (*p* = 0.046). In addition, the number of total CMBs and lobar CMBs was significantly higher in patients with BPS (*p* = 0.001 and 0.001). Receiver operating characteristic curves for BPS showed that for modified CAA-SVD, a cutoff score of 3.5 showed 46.7% sensitivity and 81.5% specificity. Meanwhile, for the total number of cerebral microbleeds (CMBs), a cut-off score of 2.5 showed 80.0% sensitivity and 77.8% specificity and for the number of lobar CMBs, a cut-off score of 2.5 showed 73.3% sensitivity and 77.8% specificity.

**Conclusion:**

Overall, patients with BPS showed worse CDRs, reduced psychomotor speed, higher modified CAA-SVD scores, larger numbers of total and lobar CMBs. We propose that severe modified CAA scores and higher numbers of total and lobar CMBs are potential risk factors for BPS in patients with mild dementia or MCI. Therefore, by preventing these MRI lesions, the risk of BPS may be mitigated.

## 1. Introduction

Following the advent of neuroimaging technology such as computed tomography and magnetic resonance imaging (MRI), the pathological changes attributed to cerebral small vessel diseases (SVD) have been detected in patients with stroke and dementia and have been reported to be correlated with both diseases (Shindo et al., 2020). SVD pathology is characterized by various etiologies affecting the small arteries, arterioles, capillaries and venules, of the brain, as well as clinical and neuroimaging features (Pantoni, 2010). The two major types of SVD are arteriolosclerosis, which is associated with hypertensive arteriopathy, and cerebral amyloid angiopathy (CAA) (Pantoni, 2010; Shindo et al., 2020). Brain MRI has been used to detect of SVD lesions, including white matter hyperintensities (WMH), lacunar infarcts, cerebral microbleeds (CMBs), cortical superficial siderosis (cSS), enlargement of perivascular spaces (PVS), and cortical microinfarcts (CMIs) (Wardlaw et al., 2019). Staals et al. proposed the total SVD score, mainly obtained for hypertensive arteriopathy, being assessed by four major MRI markers including lacunar infarct, CMBs, basal ganglia (BG)-PVS, and WMH (Klarenbeek et al., 2013, Staals et al., 2014). Charidimou et al. developed an SVD scoring system for CAA evaluated with four major MRI markers of CAA including lobar CMBs, cSS, centrum semiovale (CSO)-PVS, and WMH (i.e., the CAA-SVD score) (Charidimou et al., 2016). Other CAA-specific imaging features include posterior-dominant anteroposterior distribution of WMH (Thanprasertsuk et al., 2014) and CMIs <5 mm in diameter localized within the cortex, predominantly in the occipital lobe (Ishikawa et al., 2020). We previously developed the modified CAA-SVD score based on these imaging features, and found that the scores significantly correlated with cognitive function in memory clinic patients (Matsuda et al., 2021).

SVD is not only associated with vascular dementia (VaD), but also with Alzheimer’s disease (AD), and both hypertensive arteriopathy and CAA play important roles in SVD pathogenesis. Mild cognitive impairment (MCI) is an early stage of cognitive impairment that includes prodromal forms of various dementias (Petersen, 2004). The behavioral and psychological symptoms (BPS) of dementia, such as depression, apathy, aggression, and sleep disturbance, not only exacerbate the health condition of patients, but also lead to several problems for caregivers (Kales et al., 2015). Moreover, BPS has been observed in patients with MCI and early stage dementia (Kishino et al., 2022). Factors such as age, years of education, nutritional and marital status, and the severity and duration of disease have all been correlated with BPS; however, the mechanisms have not yet been clarified (Kishino et al., 2022).

Previous reports have shown that all SVD scores are associated with cognitive function. However, the association between the BPS of dementia or MCI and the SVD score remains unclear. In this study, we prospectively investigated the relationships between the presence of BPS and SVD scores through SVD imaging and neuropsychological assessment in patients at our memory clinic.

## 2. Methods

### 2.1. Patients

In total, 42 patients consulted with our hospital memory clinic between February 2017 and July 2019 (Matsuda et al., 2021). All procedures followed the Clinical Study Guidelines of the Ethics Committee of Mie University Hospital and were approved by the internal review board (registration number: 1596). A comprehensive description of all procedures was provided to the patients, and written informed consent was obtained directly from them or their caregivers. Every patient was examined by a neurologist with sufficient experience assessing patients with dementia.

We collected data from patients who fulfilled the following inclusion criteria: (1) consulted with our hospital’s memory clinic, (2) underwent neuroimaging examinations using 3 T MRI, (3) completed neuropsychological assessments, and (4) had a global clinical dementia rating (CDR) score of 0.5 or 1.0 at enrollment. Neuropsychological and CDR assessments were performed within 3 months of MRI. No neurological events were observed during these tests or in MRI. The presence of BPS, including delusions, hallucinations, agitation/aggression, depression/dysphoria, apathy, elation/euphoria, anxiety, disinhibition, irritability/lability, and aberrant motor behavior (Ikeda et al., 2004), was assessed through interviews with caregivers and by medical records.

Patients were diagnosed with MCI if they met the following criteria: (1) memory complaints, (2) normal activities of daily living, (3) normal general cognitive function, (4) abnormal memory for age, and 5) no history of dementia. MCI was classified into amnestic type (aMCI) or non-amnestic type (naMCI) depending on the presence or absence of memory impairment, respectively (Petersen, 2011). The mean global CDR score was 0.5. Diagnoses were assigned in accordance with the National Institute on Aging–Alzheimer’s Association (NIA-AA) guidelines (McKhann et al., 2011).

### 2.2. Neuropsychological assessments

All participants underwent the following tests to assess cognitive status: mini-mental state examination (MMSE) (Mori et al., 1985), Raven’s Coloured Progressive Matrices (RCPM) (Raven, 1962), Rivermead Behavioral Memory Test (RBMT) (Wilson et al., 1989), including a standard profile score and a screening score, Mie constructional apraxia scale (MCAS) (Satoh et al., 2016), verbal fluency, and trail making test (TMT) -A/-B (Abe et al., 2004). The verbal fluency test consisted of category and letter domains. In the category verbal fluency task, participants were asked to name as many animals as possible in 1 min. In the letter verbal fluency task, participants were asked to name as many objects as possible in 1 min, beginning with each of the following four phonemes: *ka*, *sa*, *ta*, and *te* (Matsuda et al., 2021). The average scores for these four phonemes were used for statistical analyses. CDR was performed by two speech therapists, and the results were evaluated through a discussion between two neurologists and three speech therapists based on the CDR determination rules (Hughes et al., 1982). Moreover, clinical dementia rating of sum boxes (CDR-SB) was used for the evaluation of dementia severity (O’Bryant et al., 2008).

### 2.3. MRI protocol

The MRI protocol used was the same as the previous study (Ii et al., 2019, Matsuda et al., 2021). Briefly, MRI studies were performed with a 3 T MRI unit (Achieva, Philips Medical System, Best, the Netherlands) using an 8- or 32-channel phased-array head coil. We used T1- and T2-weighted images and 3D-fluid attenuated inversion recovery (FLAIR) images for the evaluation of WMH, lacunar infarcts, and PVS. Susceptibility-weighted image (SWI) sequences were used for the detection of MBs and cSS. Using 3D-double inversion recovery (DIR) and 3D-FLAIR allowed for the detection of CMIs.

Details of the 3D-DIR protocols were as follows: field of view, 250 mm; matrix, 208 × 163 (256 × 256) after reconstruction; in plane resolution, 0.98 mm × 0.98 mm; section thickness, 0.65 mm with over contiguous slice; TSE factor 173; repetition time (ms)/echo time (ms), 5,500/247; long inversion time (ms)/short inversion time (ms), 2,550/450; number of signals acquired, two; and acquisition time, 5 min 13 s.

The SWI details were as follows: field of view, 230 mm; matrix, 320 × 251 (512 × 512) after reconstruction; in-plane resolution, 0.45 mm × 0.45 mm; section thickness, 0.5 mm with over contiguous slice; repetition time (ms)/echo time (ms), 22/11.5 (in-phase), 33 (shifted); number of signals acquired, one; flip angle 20°; and acquisition time, 5 min 45 s. 3D-FLAIR imaging was obtained in a sagittal direction, and then the axial and coronal images were reconstructed. The 3D-FLAIR details were as follows: field of view, 260 mm; matrix, 288 × 288 (364 × 364) after reconstruction; in-plane resolution (0.68 × 0.67 mm); section thickness, 1 mm with 0.5 mm overlap; no parallel imaging; repetition time (ms)/echo time (ms), 6,000/400; inversion time, 2,000 ms; number of signals acquired, two; and acquisition time, 5 min 12 s.

### 2.4. SVD scores

SVD scores were evaluated as previously described (Matsuda et al., 2021). The total SVD score was evaluated as follows; 1 point was awarded for the severity of each of the 4 markers (lacunar infarcts, CMBs, basal ganglia-PVS, and WMH), with a minimum score of 0 and a maximum score of 4 (Klarenbeek et al., 2013). The CAA-SVD score was calculated as sum of points for the severity of each of the 4 markers (lobar MBs, cSS, centrum semiovale PVS (CSO-PVS), and WMH) with a minimum score of 0 and a maximum score of 6 (Charidimou et al., 2016). The modified CAA-SVD scores includes additional imaging markers, the presence of posteriorly dominant WMH and CMIs related to CAA, and CAA [posterior score of 0 and, maximum score of 8 (Matsuda et al., 2021)]. CMBs were counted according to the Microbleed Anatomical Rating Scale (MARS) (Gregoire et al., 2009) using SWI. All scores were independently assessed by two neurologists (YI and AS).

### 2.5. Statistical analysis

Patient date relevant to sex, medical history, medication, CDR, and possible or probable Boston criteria version 2.0 (Charidimou et al., 2022) in patients with and without BPS were analyzed using chi-squared (χ2) tests. Differences in demographic variables and results from the SVD scores (each point) and neuropsychological assessment between the two groups (with and without BPS) were analyzed using the Mann–Whitney U test. Clinical and radiological characteristics were presented as numbers with percentages and means ± standard deviations. Receiver operating characteristic (ROC) curves were constructed to assess the sensitivity and specificity of MRI findings and the SVD scores in predicting BPS. The SPSS Statistics 24 software package was used to perform the descriptive statistical analyses. The R statistical package (version 4.2.2) was used to perform for the comparing the areas under ROC curves between total SVD score and modified CAA-SVD score. Statistical significance was set at *p* < 0.05.

## 3. Results

### 3.1. Study patients

A total of 42 patients enrolled in this study. The mean age was 75.3 years old for all patients and 23 patients were male. The mean educational history was 11.9 years. Overall 30 patients had MCI (CDR =0.5) while 12 patients had dementia (CDR =1.0). All 12 patients had dementia (CDR = 1.0). All 12 patients with CDR 1.0 were diagnosed with AD, while 20 with CDR 0.5 had amnestic MCI, and the remaining 10 with CDR 0.5 had non-amnestic MCI.

The demographic and clinical data of the patients with and without BPS are shown in Table 1. Of the 42 patients, 15 showed BPS and symptoms were classified as follows; apathy 11 (73.3%), agitation/aggression 2 (13.3%), delusions 1 (6.7%), and depression/dysphoria 1 (6.7%). 6 patients with BPS (40.0%) and 24 without (88.9%) had a CDR of 0.5, while 9 patients with BPS (60.0%) and 3without (11.1%) had a CDR of 1.0 (*p* = 0.001).

**Table 1:** Characteristics in the 42 patients with mild cognitive impairment or mild dementia.

	All	With BPS	Without BPS	*p* value
	*n* = 42	*n* = 15	*n* = 27	
Mean age (years)	75.3 ± 9.1	71.9 ± 12.1	77.1 ± 6.4	0.399
Male sex (*n*, %)	23 (54.7%)	5 (33.3%)	18 (66.7%)	0.055
Educational history (years)	11.9 ± 2.3	11.7 ± 1.2	12.0 ± 2.8	0.739
Clinical dementia rating (CDR)				0.001*
CDR 0.5	30 (71.4%)	6 (40.0%)	24 (88.9%)	
CDR 1.0	12 (28.6%)	9 (60.0%)	3 (11.1%)	
Clinical dementia rating of sum boxes	3.2 ± 1.7	4.2 ± 2.0	2.6 ± 1.2	0.006*
Diagnosis of cerebral amyloid angiopathy				
Possible or probable for Boston criteria version 2.0 (*n*, %)	32 (76.2)	12 (80.0%)	20 (74.1%)	0.487
Past history				
Hypertension (*n*, %)	22 (52.4%)	7 (46.7%)	15 (55.6%)	0.744
Dyslipidemia (*n*, %)	11 (26.2%)	3 (20.0%)	8 (29.6%)	0.715
Diabetes mellitus (*n*, %)	5 (11.9%)	1 (6.7%)	4 (14.8%)	0.640
Stroke (*n*, %)	5 (11.9%)	3 (20.0%)	2 (7.4%)	0.142
Smoking (*n*, %)	11 (26.2%)	4 (36.4%)	7 (25.9%)	1.000
Medication				
Anti-hypertensive agent (*n*, %)	7 (16.7)	0 (0.0%)	7 (25.9%)	0.038*
Statin (*n*, %)	6 (14.3)	1 (6.7%)	5 (18.5%)	0.395
Anti-thrombotic agent (*n*, %)	8 (19.1)	2 (13.3%)	6 (22.2%)	0.689

BPS, Behavioral and psychological symptoms.

*Differences with *p* < 0.05 was considered statistically significant.

CDR-SB was significantly higher in patients with BPS (4.2 ± 2.0) than in those without BPS (2.6 ± 1.2) (*p* = 0.006). Possible or probable CAA was diagnosed in 32 patients and there was no significant difference between the patients with and without BPS. No significant difference in past medical history, including hypertension, dyslipidemia, diabetes mellitus, stroke, and smoking, was noted between the two groups, but the use of antihypertensive agents was significantly higher in patients without BPS (25.9%) than in those with BPS (0.0%) (*p* = 0.038).

### 3.2. Factors associated with neuropsychological tests

There were no significant differences in MMSE, RCPM, RBMT, verbal fluency, and MCAS scores between patients with BPS and those without BPS (Table 2). The time taken to complete TMT set-A was significantly longer in patients with BSP (308.9 s ± 148.5 s) than in those without BPS (230.3 s ± 155.1 s) (*p* = 0.037). TMT set-B did not show any significant difference. All patients finished the TMT set-A; however, 11 of all 42 patients (26.2%), including 5 of the 27 patients without BPS (18.5%) and 6 of the 15 patients with BPS (40.0%), could not follow through to the end of the TMT set-B.

**Table 2:** Comparison of neuropsychological tests.

	All	With BPS	Without BPS	*p* value
	*n* = 42	*n* = 15	*n* = 27	
MMSE score	25.2 ± 2.4	24.7 ± 2.5	25.5 ± 2.3	0.296
**RCPM**				
Score	24.2 ± 5.7	25.9 ± 5.1	23.4 ± 5.9	0.235
Time (s)	4,401.0 ± 198.3	476.1 ± 212.0	421.3 ± 192.2	0.505
**RBMT**				
Profile score	11.5 ± 5.5	10.1 ± 5.7	12.3 ± 5.3	0.142
Screening score	4.5 ± 2.8	3.7 ± 2.8	5.0 ± 2.7	0.127
**TMT**				
Set-A time (s)	257.1 ± 155.7	308.9 ± 148.5	230.3 ± 155.1	0.037*
Set-B time (s)	264.6 ± 95.6	279.3 ± 110.3	258.5 ± 91.0	0.716
**Verbal fluency**				
Animal (n)	10.9 ± 3.9	10.6 ± 4.2	11.1 ± 3.9	0.626
Letter (n)	5.0 ± 1.7	4.8 ± 2.1	5.2 ± 1.5	0.327
**MCAS**				
Score	3.3 ± 1.7	2.8 ± 1.5	3.6 ± 1.8	0.383
Time (s)	49.6 ± 37.4	47.0 ± 38.1	51.0 ± 37.6	0.351

BPS, Behavioral and psychological symptoms; MMSE, Mini-mental state examination; RCPM, Raven’s Colored Progressive Matrices; RBMT, Rivermead Behavioral Memory Test; TMT, Trail making test; MCAS, Mie Constructional Apraxia Scale.

*Differences with *p* < 0.05 was considered statistically significant.

### 3.3. Comparison of small vessel disease MRI findings

**Figure 1:**
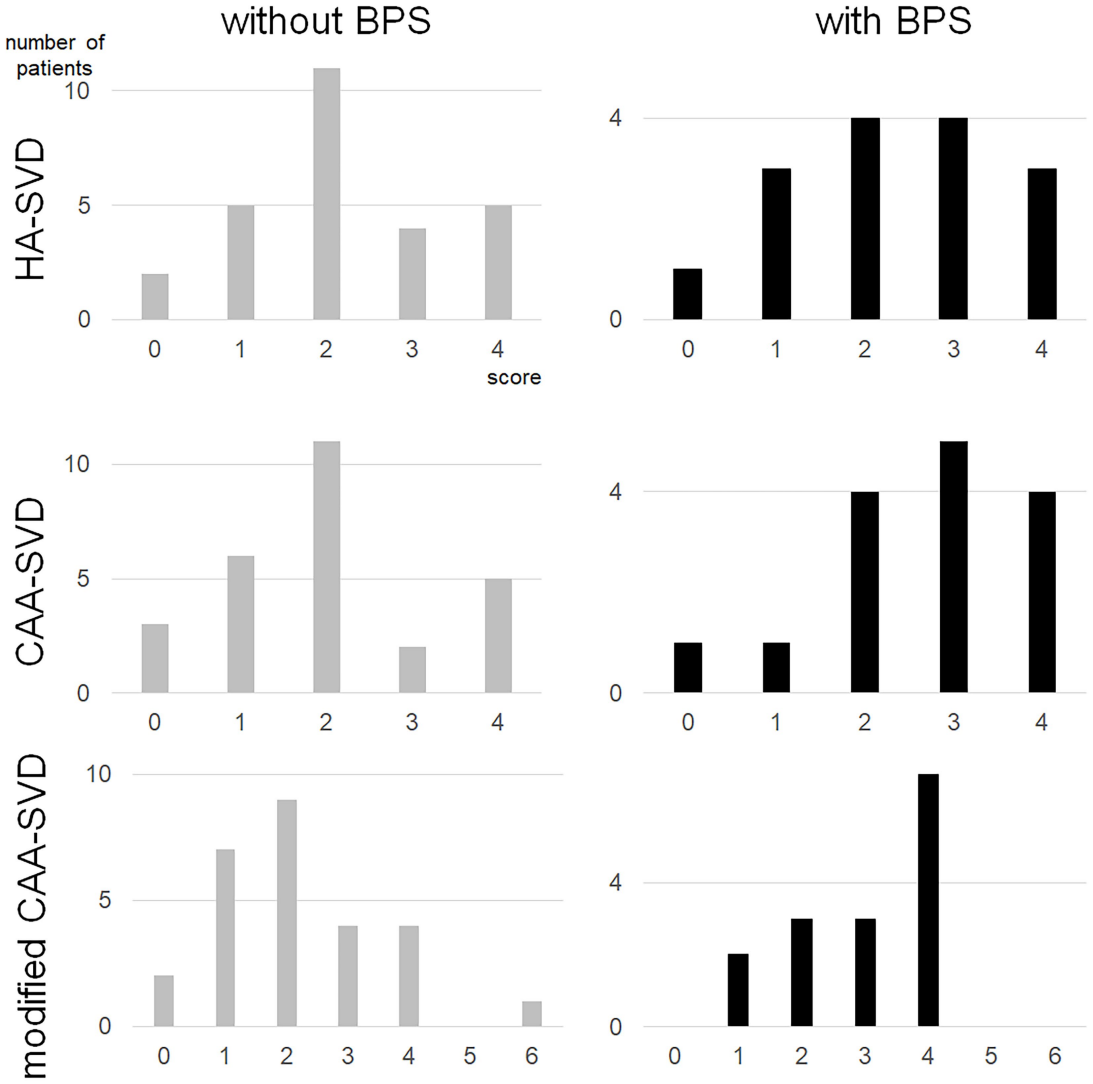
Distribution of each small vessel disease score for patients with BPS and without BPS. BPS, behavioral and psychological symptoms; CAA, cerebral amyloid angiopathy; SVD, small vessel disease.

The number of lacunar infarcts, CMIs, cSS and the severity of periventricular and deep WMHs, CMIs were not associated with BPS (Table 3). Although the number of deep CMBs did not show significant differences, the total number of CMBs and the number of lobar CMBs were significantly higher in patients with BPS than in those without BPS (*p* = 0.001 and 0.001, respectively). Regarding each SVD score, there was no significant difference in the total SVD score (*p* = 0.745) or each point of the MRI findings. The CAA-SVD score tended to be higher in patients with BPS (2.7 ± 1.2) than in those without BPS (2.0 ± 1.2) (*p* = 0.096), while the point of lobar CMBs was significantly higher in patients with BPS (1.2 ± 0.7) than in those without BPS (0.6 ± 0.8) (*p* = 0.029). Although there was no significant difference in the posterior distribution of WMH or CMI due to CAA, the modified CAA-SVD score was significantly higher in patients with BPS (3.0 ± 1.1) than in those without BPS (2.2 ± 1.4) (*p* = 0.046) (Figure 1).

**Table 3:** Comparison of small vessel disease MRI findings.

	All	with BPS	without BPS	*p* value
	*n* = 42	*n* = 15	*n* = 27	
**SVD MRI findings**				
Number of lacunar infarcts (*n*)	0.6 ± 1.2	0.7 ± 1.6	0.5 ± 1.0	0.544
**WMH**				
Periventricular (Fazekas)	1.9 ± 1.2	1.3 ± 1.2	1.7 ± 1.1	0.371
Deep (Fazekas)	1.5 ± 1.1	1.7 ± 1.3	2.0 ± 1.1	0.489
Total Number of CMBs (*n*)	5.1 ± 9.0	5.9 ± 6.9	4.7 ± 10.1	0.001*
Number of Lobar CMBs (*n*)	4.5 ± 8.1	4.9 ± 5.8	4.1 ± 9.3	0.001*
Number of Deep CMBs (*n*)	0.7 ± 1.8	1.0 ± 2.4	0.5 ± 1.1	0.442
Number of CMIs (*n*)	1.0 ± 2.4	0.1 ± 0.3	0.1 ± 0.3	0.642
Number of cSS (*n*)	1.0 ± 2.4	0.1 ± 0.4	0.1 ± 0.6	0.277
**SVD score and each point**				
Total-SVD score	2.3 ± 1.2	2.3 ± 1.2	2.2 ± 1.2	0.745
Lacunar infarcts (0–1)	0.3 ± 0.5	0.3 ± 0.5	0.3 ± 0.5	1.000
CMBs (0–1)	0.7 ± 0.5	0.9 ± 0.4	0.6 ± 0.5	0.163
Basal ganglia PVS (0–1)	0.6 ± 0.5	0.5 ± 0.5	0.6 ± 0.5	0.547
WMH (0–1)	0.6 ± 0.5	0.6 ± 0.5	0.6 ± 0.5	0.852
CAA-SVD score	2.3 ± 1.3	2.7 ± 1.2	2.0 ± 1.2	0.096
Lobar CMBs (0–2)	0.8 ± 0.8	1.2 ± 0.7	0.6 ± 0.8	0.029*
cSS (0–2)	0.1 ± 0.3	0.1 ± 0.4	0.0 ± 0.2	0.251
Centrum semiovale PVS (0–1)	0.7 ± 0.5	0.7 ± 0.5	0.7 ± 0.5	0.841
Modified CAA-SVD score	2.5 ± 1.3	3.0 ± 1.1	2.2 ± 1.4	0.046*
Posterior distribution of WMH (0–1)	0.2 ± 0.4	0.3 ± 0.5	0.1 ± 0.3	0.200
CMIs due to CAA (0–1)	0.1 ± 0.3	0.1 ± 0.3	0.1 ± 0.3	0.642

BPS, Behavioral and psychological symptoms; CAA, Cerebral amyloid angiopathy; CMBs, Cerebral microbleeds; CMIs, Cortical microinfarcts; cSS, Cortical superficial siderosis; MRI, Magnetic resonance imaging; PVS, Enlargement of perivascular spaces; SVD, small vessel disease; WMH, White matter hyperintensities.

*Differences with *p* < 0.05 was considered statistically significant.

### 3.4. ROC curves for BPS

**Figure 2:**
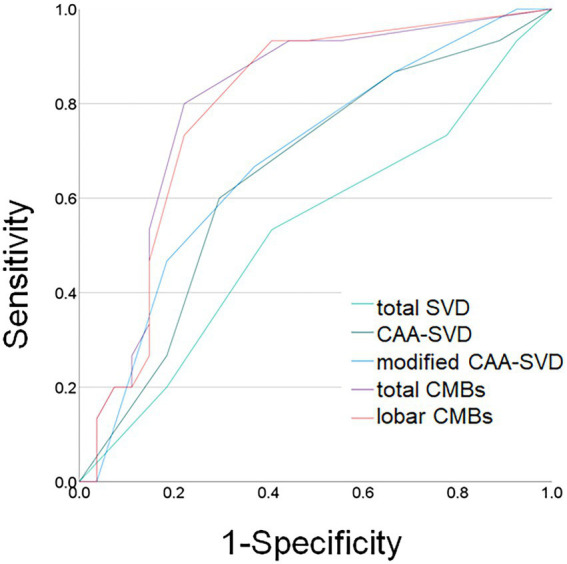
Receiver operating characteristic (ROC) curves for behavioral and psychological symptoms and MRI findings. Light green line: total small vessel disease (SVD) score; dark green line: cerebral amyloid angiopathy (CAA) SVD score; blue line: modified CAA-SVD score; purple line: total number of cerebral microbleeds (CMBs); orange line: number of lobar CMBs.

Figure 2 shows the ROC curve analysis of the patients with and without BPS. For the total SVD scores, the area under the curve (AUC) was 0.530, with a cut-off score of 2.5 and showed 53.3% sensitivity and 59.3% specificity. For CAA-SVD scores, the AUC was 0.652, with a cut-off score of 2.5 and showed 60.0% sensitivity and 70.4% specificity. For the modified CAA-SVD scores, the AUC was 0.683, with a cut-off score of 3.5 and showed 46.7% sensitivity and 81.5% specificity. The total number of CMBs, AUC was 0.800, with a cut-off score of 2.5 and showed 80.0% sensitivity and 77.8% specificity. For the number of lobar CMBs, the AUC was 0.791, with a cut-off score of 2.5 and showed 73.3% sensitivity and 77.8% specificity. The areas under ROC curves of total SVD and modified CAA-SVD scores were 0.538 and 0.690 each, and showed significantly difference (*p* = 0.015).

## 4. Discussion

This study revealed the several associations between neuropsychological and SVD MRI findings and the presence of BPS in patients with MCI or mild dementia in our memory clinic. First, the patients with BPS showed worse CDR and CDR-SB. Second, the patients with BPS required more time to complete the TMT set-A. Third, the SVD MRI findings, such as the modified CAA-SVD score and total CMBs and lobar CMBs, were associated with the presence of BPS. ROC curve analysis revealed that modified CAA-SVD scores greater than 3.5 were associated with the presence of BPS. Moreover, more than 2.5 each of the total CMBs and lobar CMBs were associated with the presence of BPS.

Patients with BPS took longer to complete the TMT set-A. A previous report showed that the BPS was associated with TMT sets -A and -B in patients with AD (García-Alberca et al., 2011). The TMT is used to evaluate visual attention, psychomotor speed, and executive function. Shi et al. reported that patients with SVD showed executive dysfunction, which was determined using the TMT score (Shi et al., 2022). Processing speed has been associated with BPS in patients with CAA and likewise, our study revealed that the time taken to complete TMT set-A was longer in patients with BPS. A previous report demonstrated that patients with CAA took a longer time in the TMT set-A time than healthy controls, and MCI due to AD showed no difference with healthy controls (Planton et al., 2017). Since 76.2% of patients in this study met the possible or probable Boston criteria version 2.0, our results were comparable to those of previous studies (Planton et al., 2017; Smith et al., 2021).

This study revealed that the patients with BPS had a higher modified CAA-SVD score and a larger number of total CMBs and lobar CMBs. Moreover, the original version of CAA-SVD score revealed a tendency for higher scores in patients with BPS. The manifestations of BPS can be influenced by various factors including patient characteristics, caregivers, and living environments (Kales et al., 2015). In particular, apathy is associated with functional deficits in medial-frontal regions and structural atrophy (Kales et al., 2015). Additionally, SVD MRI findings can present as a variety of symptoms, such as apathy, depression, cognitive impairment, and parkinsonism (Tomimoto, 2015). Cai et al. showed the association between MRI findings and apathy, such that lacunar infarcts, CMBs, and severe WMH were detected in patients with apathy (Cai et al., 2022). Hypertension and blood pressure fluctuations can cause damage to brain connections and are associated with white matter disruption, apathy and depression (Hachinski et al., 2022). Moreover, both hypertension and CAA have been associated with apathy (Smith et al., 2021), indicating that small-vessel lesions may also be associated with apathy. In this study, 15 of the 42 patients (35.7%) showed some level of BPS, with 11 of the 42 patients presented with apathy (26.2%). Thus, 70% of the patients with BPS were apathetic. In previous reports, almost 50% of patients with dementia and only 12.1% of patients without dementia present with apathy (Savva et al., 2009). Given that our study assessed patients with MCI or mild dementia, an intermediate value for the prevalence rate of apathy could be determined. The modified CAA-SVD score had more predictability than the original version of the CAA-SVD score in this study. Although for each SVD MRI marker, the CMIs due to CAA and posterior dominant distribution of WMH did not show any significance, a previous report revealed that microinfarcts in the cerebral cortex associated with mild AD and posterior WMH might be a marker of early-stage AD (Liu et al., 2020, Pålhaugen et al., 2021). Both the CMIs and posterior WMH might have additionally contributed to AD, and this might affect the modified CAA-SVD score benefit.

ROC curve analysis between patients with and without BPS revealed that the risk of BPS may be associated with a modified CAA-SVD score > 3.5 points. In addition, both the numbers of lobar and total CMBs may also be linked to BPS. A previous report revealed that white matter damage is correlated with BPS in AD (Makovac et al., 2016). Moreover, total SVD score has been associated with a component of BPS in SVD patients (Zhào et al., 2021). Our findings support these studies such that higher numbers of both lobar and total CMBs were associated with BPS in memory clinic patients. Moreover, the modified CAA-SVD score may be a useful tool for the evaluation of BPS. Although a previous report showed an association between the total SVD score and BPS, our results revealed that only the modified CAA-SVD score was associated with BPS. Perhaps, this may be due to differences in patient characteristics.

Our study had several limitations. First, we assessed the existence of BPS only through clinical interviews and medical records; hence, the quantification of BPS was not evaluated using scales such as the neuropsychiatric inventory (NPI), which has been previously associated with SVD score (Zhào et al., 2021). Second, the previous reports about the association between SVD and apathy mainly focused on hypertensive small vessel disease and thus may not be relevant to cerebrovascular diseases (Saji et al., 2021; Zhào et al., 2021). Third, although more than half of our patients met the criteria for CAA, a recent report has shown that neuropathological findings of CAA were not associated with neuropsychological symptoms (Gibson et al., 2022); however, their result might be due to the number of cases with vascular lesions in their study, as other reports clearly show that neuropsychological symptoms are common in CAA (Smith et al., 2021). Fourth, we could not obtain longitudinal data related to BPS, neuropsychological tests, or MRI findings. If these were evaluated, the manifestation of BPS may be clearly revealed in patients with MCI or mild dementia. Fifth, our result showed worse CDR for patients in the BPS group. Although there was no significant difference in each SVD score between the CDR 0.5 and 1.0 groups, our sample size may have been small in this study and there remains a possibility that these patients were simply representing the progressed disease. Moreover, we could not analyze a multivariate analysis for the reason of the smaller number of subjects and the number of explanatory variables. Finally, although our data revealed that the use of antihypertensive agents was significantly higher in patients without BPS, the reason for this remains unclear and the duration of antihypertensive use was not considered. Moreover, we could not evaluate blood pressure in all 42 patients. Among patients whose blood pressure could be evaluated, the mean systolic blood pressure in patients with BPS was 123.0 ± 11.7 mmHg, and 134.2 ± 11.7 mmHg in patients without BPS, and there was no significant difference (*p* = 0.400). Although a recent study showed that medications used in the treatment of hypertension reduce cognitive decline (Cunningham et al., 2021), the prevalence of patients with BPS taking antihypertensive agents remain uncertain. Perhaps, antihypertensive agents have a neuroprotective effect on vascular lesions in patients with mild dementia or MCI. In addition, a previous meta-analysis demonstrated that antihypertensive treatment could contribute to WMH extension, although CMBs with antihypertensive agents have yet to be elucidated (Su et al., 2021). Further study of SVD treatment is warranted.

## 5. Conclusion

This study revealed the characteristics of patients with BPS-related MCI and mild dementia, and the association between MRI and neuropsychological findings. We found that patients with BPS had worse CDR, higher modified CAA-SVD scores, larger numbers of total and lobar CMBs, and reduced psychomotor speed. We propose cut-offs for severe modified CAA scores and higher numbers of total and lobar CMBs as potential MRI risk markers for BPS. Therefore, it is possible that by preventing these MRI lesions, the risk of BPS can be mitigated.

## Data availability statement

The datasets presented in this article are not readily available because the raw data supporting the conclusions of this article are available from the corresponding author, upon reasonable request. Requests to access the datasets should be directed to Akihiro Shindo, a-shindo@med.mie-u.ac.jp.

## Ethics statement

The studies involving human participants were reviewed and approved by Ethics Committee of Mie University Hospital. The patients/participants provided their written informed consent to participate in this study.

## Author contributions

MS: draft of manuscript, and acquisition of data. KaM and YI: manuscript revision, data acquisition, study concept and design, and analysis. K-iT: statistical analysis. NN, YH, HI, HM, KeM, and MM: data acquisition and interpretation. HT and AS: revision of the manuscript, study concept and design, and study supervision.

## Funding

This research was supported by KAKENHI Grant-in-Aid for Scientific Research (C Number 21 K07433 and 22 K17587).

## Conflict of interest

The authors declare that the research was conducted in the absence of any commercial or financial relationships that could be construed as potential conflicts of interest.

## Publisher’s note

All claims expressed in this article are solely those of the authors and do not necessarily represent those of their affiliated organizations, or those of the publisher, the editors and the reviewers. Any product that may be evaluated in this article, or claim that may be made by its manufacturer, is not guaranteed or endorsed by the publisher.
